# The Indications for and against Enucleation

**Published:** 1887-12

**Authors:** W. Cheatham

**Affiliations:** Louisville, Ky.; Lecturer on Diseases of Eye, Ear, Throat and Nose, University of Louisville; Eye, Ear, Throat and Nose Physician, Louisville City Hospital and Masonic Widows and Orphans’ Home and Infirmary


### THE INDICATIONS FOR AND AGAINST ENUCLEATION.

BY W. CHEATHAM, M. D.,

LECTURER ON DISEASES OF EYE, EAR, THROAT AND NOSE, UNIVERSITY OF LOUISVILLE; EYE, EAR, THROAT AND NOSE PHYSICIAN, LOUISVILLE CITY HOSPITAL AND MASONIC WIDOWS AND ORPHANS' HOME AND INFIRMARY. READ BEFORE THE LOUISVILLE SURGICAL SOCIETY, OCTOBER 3RD, 1887.

---

Most physicians look upon the enucleation of an eye as a trivial matter, and seem to think the indications for such a procedure are so well marked as to render an error impossible; there are, on the contrary, many close, fine points for the operator to study, and to which he can devote much hard thinking with profit. The operation known as enucleation of an eye is considered by a majority of the profession as devoid of danger and as being simple enough for any practitioner of medicine to undertake. And just here I will refer in a few words only to the substitutes for enucleation, one of which is evisceration; by this is meant the emptying of the globe of its contents, leaving the muscles and sclera to form a stump; it has not as yet given very satisfactory results. Again, neurotomy has been tried with like failure; so I believe there is no procedure that will displace enucleation; occasionally this has proven a failure, the wound having to be re-opened and some confined nerve released, or the optic nerve cut off farther back. Of course whenever a malignant growth of the globe is present the sooner the eye is removed the better.

Here (some say even not here) is one part of the body, if an early enucleation can be done, there may be no return of a malignant growth. I know of a patient now, both of whose eyes were enucleated about twelve years ago with no return of the trouble ; such cases are rare. If the disease has broken through the sclera, enucleation does no good, except, probably, if the disease should recur it may do so not in the orbit or about the head, but in some more distant organ, rendering death less painful and more rapid, and life less disgusting to both patient, relatives and friends. This is an argument frequently used by me when there is some hesitation on the part of parties concerned. I am sure it would be more pleasant to any of us to have such a growth attack some internal organ than attack the face. There is often difficulty, as you all understand, in diagnosing the character of tumors. I have had in the last year or so three cases of growth of the iris and ciliary body, which have gone the rounds in this city, Cincinnati and New York, some agreeing with me as to their character, but such men as Knapp and Williams differing with me. But one of these have I been able to persuade to have the eye enucleated, a section of which I exhibit to-night. The growth in this case showed but little above the edge of the pupil. You can easily see how much of it was hidden. It is a *melano-sarcoma.*

In the consideration of this subject of enucleation, though the greatest point of interest is what is known as preventive enucleation of injured eyes; or, in other words, is preventive enucleation a justifiable operation ?

Mauthner's creed is as follows: " It (that is enucleation) may be performed as a preventive. It must be performed in the stage of irritation; it cannot be performed in iritis serosa or iritis plastica; it can be performed in irido-cyclitis plastica, provided the eye causing sympathy is totally blind, but not in a state of violent irritation."

The objections to enucleation are many, a few of the most important of which only I will mention.

Death has followed as the result of the enucleation of an eye, especially if panophthalmitis be present; meningitis is the chief complication here. Death under such conditions has also been the result of hemorrhage. I myself have had two very severe cases of hemorrhage follow enucleation, in one of which our President assisted me. The second case was in the person of a very feeble old lady. Again, I have had a very serious meningeal complication follow the enucleation of a recently injured eye, in which the tissues of the orbit were greatly involved.

The Germans say not enucleate under such conditions. Von Graefe had two deaths follow such a procedure. The English say enucleate—there is no danger. I have just had a case bearing on this point, in New Albany, in which I waited until the panophthalmitis subsided. Another objection is the disfigurement or mutilation. There is no greater deformity to a face than sunken lids and an empty socket. It is true it is very much corrected by wearing a glass eye, yet this is an expensive luxury; many cannot afford it. Glass eyes break easily, and the secretions of the socket erode them so as to render at least one a year a necessity. I can in no other way give my views in reference to enucleation than by citing a series of cases I have had in the last three or four weeks. Mr. H., Clarksville, Tenn. Gun-shot wound of left eye; sight of injured eye O; other eye perfect. Man of moderate means and not very intelligent. Cannot stay here to have eye treated. Enucleation advised, first, because the man could not afford to lose the time to have the eye treated. The eye was blind. His intelligence was not, in my estimation, enough to realize symptoms of sympathetic irritation. Miss G., of New Albany, could not afford to lose the time necessary for treatment. Mr. B., of this city, the same. Mr McK., New Providence, Ind., had a small stump in right eye socket. In left eye there was serious irido-cyclitis. Vision left 2-5. Stump removed. Patient put on hyd. bichlor. and pilocarpin. Vision in a few weeks 20-20 or perfect. Mrs. K., glaucoma hemorrhagica: v=0; eye enucleated on account of pain. Mr. N., Jeffersonville Ind.

Piece of steel in left eye for 8 weeks v=o: vision of fellow-eye=O. Enucleation not advised, it being too late. Some months ago, I had three cases similar to the first reported gun-shot wounds. All were gentleman who had time and means to wait and go under proper treatment. Two of the eyes were saved plump, with but little deformity, but no vision. No. three got vision 2-5. It will be observed that not only the severity and character of the injury, but the mental and financial condition of the patient are important factors in such cases.

Again, the condition of the good eye is of great importance. It is said that sympathetic inflammation cannot occur before the fourth week. Some say it may occur suddenly. The weight of authorities say not. I believe we always have warning of what is known as sympathetic irritation, such as occasional blurring of vision, hyperæsthetic retina, as indicated by photophobia, eyes tiring easily, phosphenes, etc. As Mauthner lays down in his creed, the injured eye must not be enucleated if the sympathetic inflammation is of the serous form. Why? Because the enucleation often transforms the serous inflammation into a plastic one, thereby increasing the danger of the fellow-eye. I will give in a few words some cases reported by Mauthner and his translators, Webster and Spalding, bearing on "Mauthner's Creed."

Hasket Derby enucleated an injured eye which had vision $\frac{1}{40}$ of perfect, the fellow suffering from *sympathetis serous iritis.* The good eye cleared up in a few weeks. A series of attacks of serous iritis followed with loss of that eye. Suppose the originally injured eye with vision $\frac{1}{40}$ had not been enucleated, what a comfort it would have been! Knapp enucleated one eye when the fellow-eye had iritis serosa. Plastic irido choroiditis followed, reducing vision to $\frac{1}{100}$, afterwards returning to $\frac{1}{10}$. The case was not seen afterwards.

Samelsohn had a boy, fourteen years old, with iritis serosa, left eye, following a blow from a piece of rubber. Vision was reduced to counting fingers with difficulty. Just here the same trouble set up in his right eye. Enucleation of left eye is advised. Patient's parents would not submit. In six weeks vision of right eye is normal, that of left eye one-half of normal. If the left eye had been enucleated, the result would have very likely been the same as that in Dr. Derby's case. So it will be observed that the path of the early preventive enucleator is not one "strewn with roses," but that of the too-late enucleator is surely one of thorns. It occurs to me that "Mauthner's Creed" is a good one. Of course no set rules can be made to govern all cases. His are, in my estimation, the most perfect that have yet been formulated.

